# ST-TriMambaUNet: A Weather Radar Echo Extrapolation-Based Spatiotemporal Sequence Prediction Network for Precipitation Nowcasting

**DOI:** 10.3390/s26144461

**Published:** 2026-07-14

**Authors:** Heng Wang, Qiang Sun, Yu Shi

**Affiliations:** 1 School of Automation and Information Engineering, Xi’an University of Technology, Xi’an 710048, China; wangheng_xaut@163.com; 2 Xi’an Huyi District Meteorological Bureau, Xi’an 710300, China; 18049229580@163.com

**Keywords:** radar echo extrapolation, precipitation nowcasting, spatiotemporal fusion attention, Mamba, channel shuffle, directional features, multi-scale convolution

## Abstract

Precipitation nowcasting plays an important role in mitigating the impacts of extreme weather events on social production and daily life. However, existing methods still face two major limitations. (1) Convolutional neural network-based methods are insufficient in modeling the temporal dependencies of radar echo sequences, which may lead to information loss in the prediction results. (2) Most existing methods enlarge the receptive field by stacking convolutional layers. This strategy makes it difficult to obtain a truly global receptive field and effectively model global dependencies, resulting in limited accuracy in heavy rainfall prediction. In addition, spatiotemporal information at different time steps is not fully integrated, and the multi-scale directional features of rainbands are often ignored. To address these issues, this paper proposes ST-TriMambaUNet, which consists of an encoder, a decoder, and a feature enhancement module. First, a Spatiotemporal Fusion Attention (STFA) was designed, including global spatial attention and temporal attention. It can effectively learn long-range spatial correlations and capture the temporal dependencies of radar echo sequences in a parallel manner. Second, a Multi-Scale Interaction Mamba (MSIM) module was developed with three branches. The first branch leverages Mamba to model global spatiotemporal dependencies with linear complexity. The second branch promotes spatiotemporal information interaction through channel shuffle and further combines Mamba to model global spatiotemporal dependencies. The third branch designs Multi-Scale Directional Convolution (MSDC) to learn the multi-scale directional features of rainbands. Finally, the features from the three branches are dynamically fused through the designed adaptive gated fusion mechanism. This enhances the model’s representation capability for strong-echo core regions and multi-scale precipitation band structures. Experimental results on two public datasets, SEVIR and CIKM, demonstrated that the proposed ST-TriMambaUNet achieved clear advantages in both overall prediction accuracy and heavy rainfall scenarios. In particular, under the high-threshold precipitation scenarios of SEVIR (160, 181, and 219), the CSI was improved by up to 10.19%. In the heavy rainfall scenario of CIKM at 40 dBZ, CSI, POD, and HSS were improved by 5.29%, 8.07%, and 4.65%, respectively.

## 1. Introduction

Precipitation nowcasting refers to rainfall forecasting over a specific region within the next 0–2 h at high spatial and temporal resolutions. It focuses on precipitation events that are characterized by short duration and high intensity, and has become one of the key technologies for disaster prevention, mitigation, and refined urban management [1]. Accurate precipitation nowcasting provides important support for practical applications such as traffic dispatching, agricultural production, and urban flood control. Therefore, it has attracted extensive attention from researchers in meteorology and computer vision [2].

At present, precipitation nowcasting methods can be broadly divided into numerical weather prediction (NWP) [3] and radar echo extrapolation-based methods [4]. For sudden precipitation nowcasting tasks, NWP usually suffers from high computational cost and limited timeliness. This makes it difficult to apply in operational scenarios [5]. Compared with NWP methods, radar echo extrapolation-based methods only rely on several historical radar echo maps. They do not require a large number of meteorological variables [6]. From an algorithmic perspective, radar echo extrapolation-based methods can be further divided into traditional extrapolation methods and deep learning-based radar echo extrapolation methods. Optical flow is a typical traditional extrapolation method. However, this type of method is usually based on the ideal assumption of pixel-intensity conservation, which is often violated in the real world [7]. Therefore, optical flow methods have obvious limitations. They cannot well characterize the intensification or weakening of radar echoes, nor can they effectively capture the initiation or dissipation of radar echoes. With the increasing accessibility of meteorological data, deep learning has emerged as a powerful data-driven technique. It shows a strong ability to learn the evolution patterns of spatiotemporal features, thereby greatly promoting the development of precipitation nowcasting.

Early studies mostly adopted autoregressive architectures that combine convolution with recurrent neural networks. Shi et al. [8] pioneered the integration of convolution with long short-term memory (LSTM) [9] and proposed ConvLSTM. This model formed an encoder-forecaster structure to jointly learn temporal and spatial information. Wang et al. [10] improved the spatiotemporal memory unit and the flow of spatiotemporal information. They proposed a zigzag spatiotemporal memory flow and a dual-memory decoupling mechanism, laying a solid foundation for subsequent studies. On this basis, PredRNN++ was further proposed [11]. By introducing Causal LSTM, this model enhanced the modeling of short-term dynamic features. It also incorporated a Gradient Highway Unit (GHU) to effectively alleviate the vanishing gradient problem. In addition, E3D-LSTM [12] introduced 3D convolutions into the recurrent structure to strengthen short-term spatiotemporal feature modeling. The above methods rely on recurrent structures to capture long-term temporal dependencies in an autoregressive manner. As a result, they cannot be computed in parallel and usually suffer from low computational efficiency and slow training speed. Moreover, they are prone to error accumulation and propagation, which may lead to blurry predictions. CNN-based methods generate future frames in a non-autoregressive manner and can avoid these problems. Therefore, to improve computational efficiency and overcome the limitations of recurrent networks in parallelism and error accumulation, researchers began to explore recurrent-free models. SimVP [13] first proposed replacing recurrent structures with a purely convolutional architecture. It used an intermediate temporal encoder to model temporal dependencies in parallel. However, its convolutional nature is still constrained by local receptive fields, which limits its ability to capture long-range temporal dependencies. Subsequently, TAU [14] further designed a parallelizable temporal attention unit. It decomposes temporal modeling into static attention and dynamic attention, enabling parallel temporal modeling.

Although a parallelizable temporal attention unit can remove the constraints of recurrent structures and better capture global temporal dependencies, the convolution-based spatial attention still relies on local neighborhood features. Thus, it is difficult to establish dependencies among global pixels. As a result, when the model handles large-scale spatial correlations, its attention responses tend to be locally biased and lack sufficient global perception. Therefore, a mechanism is needed to capture both the temporal dependencies among radar echoes and the long-range spatiotemporal dependencies in a parallel manner.

Convolutions are effective at learning spatial features from images. They are suitable for modeling the spatial correlations of radar echo maps. Based on the UNet [15] architecture, various methods have been developed. SmaAt-UNet [16] significantly reduced the number of parameters while maintaining precipitation prediction accuracy comparable to that of UNet. On this basis, SAR-UNet [17] introduced residual connections in parallel with depthwise-separable convolution within the SmaAt-UNet framework to alleviate gradient vanishing. SSA-UNet [18] further improved forecasting performance while reducing the number of parameters. These UNet-based networks rely on convolutions to learn spatial features. However, convolution kernels are local operators, and each convolutional layer can only learn features from a limited receptive field. As a result, these methods cannot obtain a truly global receptive field. It should also be noted that these methods often suffer from insufficient spatiotemporal information interaction across different time steps, which limits prediction accuracy. Since the self-attention mechanism can obtain a global receptive field, a series of methods based on UNet and Transformer [19] have been developed. For example, AA-TransUNet [20] introduced self-attention into the bottleneck layer to improve the capture of global dependencies in radar echoes. To better learn global features and further improve the prediction accuracy of heavy rainfall, Rainformer [21] was built upon Swin Transformer [22] and designed a feature-extraction-balancing unit. This unit effectively considers both local and global feature learning. However, self-attention usually has high computational complexity. In addition, redundant propagation of spatiotemporal information may occur through skip connections. To address these issues, HTLA [23] designed a cross-channel lightweight attention mechanism and Gaussian pooling-based skip connections. RainHCNet [24] addressed the limitation that existing convolutional networks mainly focus on local high-frequency information while ignoring low-frequency components. It designed a hybrid channel-spatial attention mechanism, thereby improving the model’s ability to predict heavy rainfall.

The above methods can obtain a global receptive field through self-attention mechanisms, thereby improving the prediction accuracy for heavy rainfall. However, self-attention usually has high computational complexity. This limits forecast timeliness in precipitation nowcasting tasks with high spatiotemporal resolution. Moreover, these methods ignore the multi-scale directional features of heavy rainfall regions, such as rainbands distributed along latitudinal and longitudinal directions. As a result, their prediction accuracy for heavy rainfall remains insufficient.

To address the above issues, this paper proposes a spatiotemporal fusion attention-enhanced tri-branch MambaUNet for precipitation nowcasting, termed ST-TriMambaUNet. A Spatiotemporal Fusion Attention (STFA) is designed. It consists of global spatial attention and temporal attention. Specifically, a global spatial attention map is first obtained by computing global pixel-wise relationships, which enables the model to capture large-scale spatial dependencies. Meanwhile, temporal attention is designed along the channel dimension. It aims to adaptively assign weights to radar echo maps at different time steps. In this way, the final output can jointly consider both spatial attention and temporal attention. In addition, a Multi-Scale Interaction Mamba (MSIM) module is designed. This module consists of three parallel branches. The first branch learns global spatiotemporal dependencies with linear computational complexity. The second branch incorporates channel shuffle [25] to divide channels into different groups. This enables spatiotemporal feature interaction across different time steps, captures broader spatiotemporal correlations, and breaks the channel independence of convolutional operations. The third branch designs a Multi-Scale Directional Convolution (MSDC) branch to capture horizontal and vertical directional features, thereby enhancing the learning of multi-scale directional patterns in heavy rainfall regions. This design preserves the ability to learn local details in the deep layers of the UNet architecture. Meanwhile, it captures global spatiotemporal dependencies with low computational complexity, which improves the prediction accuracy for heavy rainfall.

Overall, the proposed method provides a new perspective for precipitation nowcasting. The main contributions are summarized as follows.

- To capture the temporal dependencies among radar echoes and long-range spatiotemporal dependencies in a parallel manner, a Spatiotemporal Fusion Attention (STFA) is designed. It enables the model to simultaneously capture long-range spatial dependencies and temporal dependencies among radar echo maps.
- To overcome the limited receptive field of convolutions in learning global dependencies, the insufficient spatiotemporal information interaction across different time steps, and the neglect of multi-scale directional features in heavy rainfall regions, a Multi-Scale Interaction Mamba (MSIM) module is proposed. It aims to strengthen spatiotemporal information interaction among different time frames and effectively learn the directional features of heavy rainfall regions, thereby improving the prediction accuracy for heavy rainfall.
- Extensive experiments were conducted on the CIKM and SEVIR datasets. The results confirmed that the proposed model can effectively improve the prediction accuracy for heavy rainfall. Both qualitative and quantitative results validated the effectiveness of the designed modules.

## 2. Method

### 2.1. Overall Network Architecture

**Figure 1:**
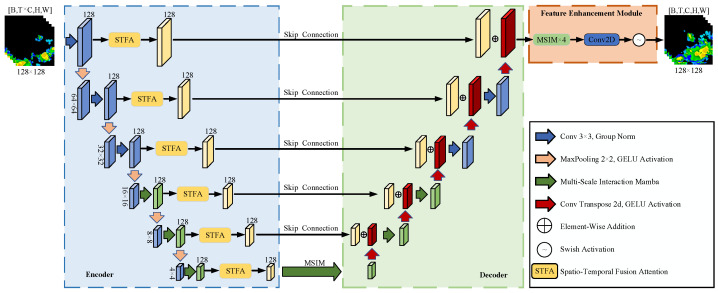
Framework of ST-TriMambaUNet.

The overall architecture of the proposed ST-TriMambaUNet is shown in Figure 1. It consists of an encoder, a decoder, and a feature enhancement module. Mamba serves as the core global spatiotemporal modeling component of the proposed ST-TriMambaUNet. Specifically, the proposed Multi-Scale Interaction Mamba (MSIM) modules are embedded in the deeper layers of the encoder and decoder, as well as in the feature enhancement module. By transforming two-dimensional spatial feature maps into sequential representations, Mamba can capture long-range spatiotemporal dependencies while maintaining linear computational complexity. In the encoder, the first three layers are composed of convolution and downsampling operations. They are used to preserve low-level local spatial details from the input radar echoes. The last three layers are composed of MSIM modules. These modules achieve a global receptive field while maintaining linear computational complexity. They also strengthen spatiotemporal information interaction across different time frames and effectively learn the multi-scale directional features of heavy rainfall regions. To compensate for the insufficient temporal dependency modeling of CNNs, the proposed Spatiotemporal Fusion Attention (STFA) is introduced into the skip connections. STFA includes global spatial attention and temporal attention. When multi-scale features learned by the encoder are transferred to the decoder, STFA enables them to consider long-range spatial dependencies. It also captures temporal dependencies among radar echoes in a parallel manner. The decoder is symmetric to the encoder. It adopts transposed convolutions for upsampling and progressively fuses the attention-enhanced features from the skip connections. This ensures sufficient integration of low-level details and high-level global information. At the end of the decoder, a feature enhancement module is further designed. It is stacked by four MSIM modules to further capture global spatiotemporal dependencies. The detailed structures and principles of the MSIM module and STFA are introduced in the following subsections.

### 2.2. Multi-Scale Interaction Mamba Module

**Figure 2:**
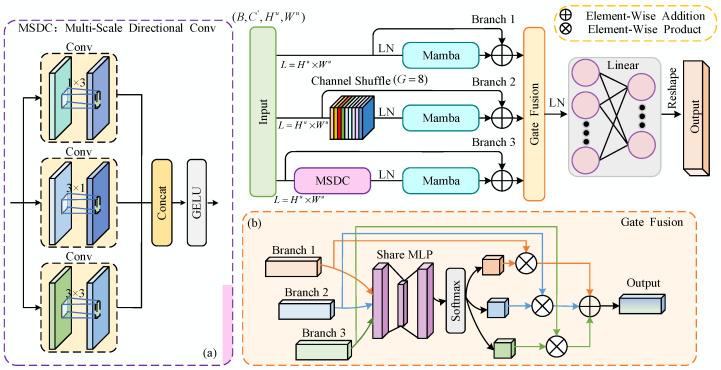
Multi-Scale Interaction Mamba (MSIM) module. (**a**) Detailed structure of the Multi-Scale Directional Conv (MSDC). (**b**) Detailed structure of the gated fusion mechanism.

Effectively learning global spatiotemporal features from radar echo images helps improve the prediction accuracy for heavy rainfall [21]. Existing convolution-based methods cannot obtain a global receptive field, while self-attention mechanisms can model global dependencies but introduce high computational complexity. In addition, existing methods often suffer from insufficient spatiotemporal information interaction across different time steps. They also ignore the multi-scale directional features of heavy rainfall regions, which still limit the prediction accuracy for heavy rainfall. To address these issues, the MSIM module is designed. As illustrated in Figure 2, Mamba serves as the core global modeling component of the three branches in the MSIM module, enabling efficient long-range spatiotemporal dependency modeling.

#### 2.2.1. Global Spatiotemporal Modeling Based on SS2D

**Figure 3:**
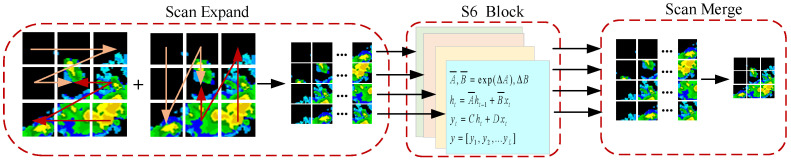
Detailed structure of SS2D.

The Visual State Space (VSS) model [26] employs the Selective Scan 2D (SS2D) mechanism to model input features along multiple spatial directions. In this way, it can effectively obtain a global receptive field. Its overall structure is shown in Figure 3.

Given an input feature *x*, the output feature *y* obtained after SS2D can be formulated as follows.

(1) $$x_{k}=\mathrm{expand}(x,k)$$

(2) $${\bar{x}}_{k}=S6(x_{k})$$

(3) $$y=\mathrm{merge}({\bar{x}}_{1},{\bar{x}}_{2},{\bar{x}}_{3},{\bar{x}}_{4})$$

where k∈{1,2,3,4} denotes four different scanning directions. expand(·) represents the scan expansion operation. S6(·) denotes the selective state space model, which is the core computational unit of Mamba. It is used to update states for one-dimensional sequences, thereby modeling long-range dependencies. merge(·) represents the scan merging operation.

Inspired by this idea, a task-specific VSS is designed for precipitation nowcasting. It should be noted that the input to MSIM is not the original two-dimensional radar image. Instead, it is a two-dimensional spatial feature map obtained after convolution and downsampling. In the three branches of this module, before the features are fed into Mamba, the two-dimensional feature maps are rearranged along the spatial dimensions into sequential representations. Each pixel is treated as a token, which corresponds to the scan expansion operation in standard VSS. These sequences are then fed into Mamba for state space modeling, fully leveraging its advantage in long-range dependency modeling. Finally, the global spatiotemporal features obtained from the three branches are fed into the designed adaptive gated fusion unit. They are then restored to a two-dimensional spatial structure, which corresponds to the scan merging operation. This entire process corresponds to the VSS designed in this paper.

#### 2.2.2. Tri-Branch Mamba Design

Precipitation nowcasting is essentially a spatiotemporal sequence prediction task based on radar echo images. The dimension of a radar echo sequence can be represented as (B,T,C,H,W), where B denotes the batch size, T denotes the number of radar echo frames, and C denotes the number of channels. For grayscale radar echo images, C is 1. H and W denote the height and width of each radar echo image, respectively. At the beginning of the network, multiple radar echo frames are used as input. In the initial stage, the temporal dimension T is merged into the channel dimension C, resulting in an input dimension of (B,T×C,H,W). For ease of subsequent description, T×C is denoted as $C^{\prime }$, where C=1. Thus, the input dimension becomes $\left(B,C^{\prime },H,W\right)$. By merging these two dimensions, convolutions can implicitly extract temporal features. Therefore, the subsequent MSIM module operates on the entire spatiotemporal feature representation.

After multiple convolutional layers, the input feature map of the MSIM module is denoted as $X\in {\mathbb{R}}^{B\times C^{\prime }\times H^{u}\times W^{u}}$, where $C^{\prime }$ denotes the number of feature channels after convolutional mapping. $H^{u}$ and $W^{u}$ denote the height and width of the feature map at the corresponding layer, respectively. Here, u∈{1,2,3,4,5,6}. When u=1, the feature map from the fourth layer of the encoder is fed into this module. When u=4, the feature map from the first layer of the decoder is fed into this module, and so forth. As shown in Figure 2, the MSIM module adopts a three-branch parallel structure.

The first branch directly models the global spatiotemporal dependencies of the input features. Specifically, the input feature map X is first flattened along the spatial dimensions into a sequence with length $L=H^{u}\times W^{u}$, and then reshaped into $X\in {\mathbb{R}}^{B\times L\times C^{\prime }}$. Next, layer normalization is applied to the sequence, which is then fed into Mamba for state space modeling. Finally, a residual connection is used to preserve the original feature information.

(4) $$X_{1}=\mathrm{Mamba}(\mathrm{LN}(X))+\mathrm{LN}(X)$$

Mamba(·) denotes the state space equation, namely S6(·) introduced in Section 2.2.1. Its core lies in state space modeling. LN(·) denotes layer normalization, and $X_{1}$ represents the output of the first branch.

The second branch introduces channel shuffle before global modeling. Specifically, the input feature map X is first divided into groups to obtain $X_{G}$. Then, channel shuffle is applied to obtain $X_{shuffle}$, enabling cross-group feature reorganization. The features are then unfolded into a sequential form. After layer normalization and Mamba-based modeling, the output is obtained through a residual connection. The purpose of this branch is not to directly rearrange the original temporal dimension. Instead, under the premise that temporal information has been embedded into intermediate channel representations, it enhances the information exchange of spatiotemporal features among different groups. This helps alleviate insufficient information interaction across different time steps.

(5) $$X_{G}=\mathrm{Group}(X,G)$$

(6) $$X_{shuffle}=\mathrm{Shuffle}(X_{G})$$

(7) $$X_{2}=\mathrm{Mamba}(\mathrm{LN}(X_{shuffle}))+X_{shuffle}$$

G denotes the number of groups, which is set to 8. Group(·) denotes the grouping operation, and Shuffle(·) denotes channel shuffle. $X_{2}$ represents the output of the second branch.

The third branch is used to enhance the extraction of local directional structures and multi-scale spatial features. First, the input feature map X is processed by the designed multi-scale directional convolutions (MSDC). Convolutions with kernels of 1×3, 3×1, and 3×3 are used to learn local directional spatial features and multi-scale directional features. Specifically, the 1×3 convolution is used to learn the movement and deformation of rainbands in the horizontal direction, while the 3×1 convolution is used to learn radar echo variations in the vertical direction. In addition, small convolution kernels can enhance the sensitivity to local edges and abrupt regions. The 3×3 convolution is used to enlarge the receptive field and supplement wider spatial context information. In this way, a balance is achieved between local fine-grained feature extraction and global dynamic modeling. The learned features are then concatenated along the channel dimension to obtain $X_{concat}$. After batch normalization and the GELU activation function, $X_{MSDC}$ is obtained, where MSDC denotes Multi-Scale Directional Conv. Then, $X_{MSDC}$ is unfolded into a sequence. After layer normalization and Mamba-based modeling, the output is obtained through a residual connection.

(8) $$X_{concat}=\mathrm{Concat}({\mathrm{Conv}}_{1\times 3}(X),{\mathrm{Conv}}_{3\times 1}(X),{\mathrm{Conv}}_{3\times 3}(X))$$

(9) $$X_{MSDC}=\mathrm{GELU}(\mathrm{BN}(X_{concat}))$$

(10) $$X_{3}=\mathrm{Mamba}(\mathrm{LN}(X_{MSDC}))+\mathrm{LN}(X_{MSDC})$$

${\mathrm{Conv}}_{1\times 3}(\cdot )$, ${\mathrm{Conv}}_{3\times 1}(\cdot )$, and ${\mathrm{Conv}}_{3\times 3}(\cdot )$ denote 1×3, 3×1, and 3×3 convolutions, respectively. BN(·) denotes batch normalization, Concat(·) denotes the concatenation operation, and $X_{3}$ represents the output of the third branch.

#### 2.2.3. Adaptive Gated Fusion Mechanism

To fully integrate the complementary features learned by the three branches, the MSIM module designs an adaptive gated fusion mechanism after multi-branch feature extraction. This mechanism dynamically adjusts the feature contributions of different branches. It can adaptively assign fusion weights to each branch at different spatial locations according to the importance of local spatiotemporal features. In this way, the fusion of multi-scale and cross-channel features is improved.

Specifically, let $X_{m}\in {\mathbb{R}}^{B\times L\times C^{\prime }}$ denote the output feature of the m-th branch, where m∈{1,2,3}. First, the features from the three branches are concatenated and fed into a shared multilayer perceptron to generate an intermediate representation of the gating weights. Then, the obtained weight vector is normalized by softmax to produce the adaptive fusion coefficients for the three branches. Finally, the three branch features are weighted and summed according to their corresponding gating coefficients, yielding the fused output feature.

(11) $$z=\mathrm{MLP}\left(\mathrm{Stack}\left(X_{1}\;,X_{2}\;,X_{3}\right)\right)$$

(12) $$\left[\alpha_{1}\;,\alpha_{2}\;,\alpha_{3}\;\right]=\mathrm{Softmax}\left(z\right)$$

(13) $$X_{fusion}\;=\sum_{m=1}^{m=3}\alpha_{m}\;⊗X_{m}\;\;$$

MLP(·) consists of two fully connected layers and a ReLU activation function. Stack(·) denotes the concatenation operation. $\alpha_{m}$ denotes the fusion weight of the m-th branch at the current spatial location, and ⊗ denotes element-wise multiplication.

Through this adaptive gated fusion mechanism, the model can dynamically adjust the fusion of multi-branch features at each spatial location. The fusion weights are adaptively generated based on the features of the three branches at each spatial position. In this way, the contribution of each branch is dynamically regulated, enabling effective complementary fusion of multi-scale and multi-directional features. The fused features are further processed by layer normalization and linear projection. They are then reshaped into a two-dimensional spatial structure. Finally, the temporal and channel dimensions are separated to obtain the final output of this module.

(14) $$\tilde{X}=\mathrm{Reshape}(\mathrm{Linear}(\mathrm{LN}(X_{fusion}\;))),\tilde{X}\in {\mathbb{R}}^{B\times T\times C\times H\times W}\;$$

### 2.3. Spatiotemporal Fusion Attention for Precipitation Nowcasting

**Figure 4:**
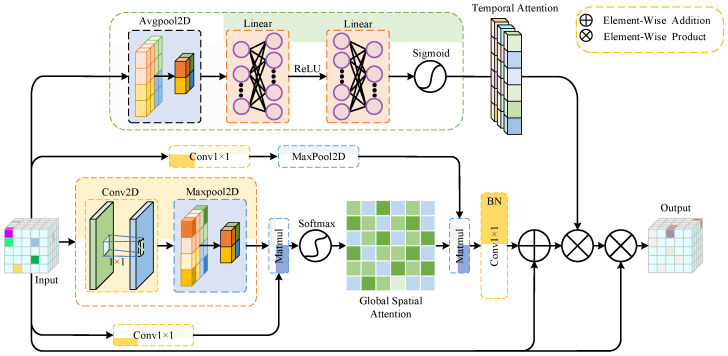
Spatiotemporal Fusion Attention (STFA).

Learning the temporal dependencies of radar echoes is crucial for improving the prediction accuracy of long sequences. However, recurrent temporal models are difficult to parallelize and are prone to error accumulation. Although CNNs have advantages in parallel computation, their ability to model long-range temporal dependencies is limited. Convolution-based temporal attention methods still rely on local neighborhood features in essence. Thus, they struggle to establish global spatiotemporal dependencies, leading to insufficient global perception in large-scale spatial modeling. Based on this, this paper proposes STFA to jointly model global spatial attention and temporal attention. Specifically, global spatial attention enables each pixel position to attend to relevant regions over the entire spatial domain. This breaks the limitation of local receptive fields in convolutions and generates a two-dimensional spatial attention map to enhance feature responses at different locations. Temporal attention adaptively adjusts the importance of different time frames through global channel weighting, further strengthening the model’s ability to capture temporal dependencies. In addition, skip connections in UNet directly concatenate encoder and decoder features. This may ignore different spatiotemporal dependencies and introduce redundant information. To address this issue, STFA is introduced into the skip connections. It enables the model to dynamically perceive the correlations of precipitation echoes in spatial distribution and temporal evolution, thereby achieving more selective spatiotemporal feature transmission. Its structure is shown in Figure 4.

#### 2.3.1. Global Spatial Attention

For the global spatial attention branch, the core idea is to generate the query vector Q, key vector K, and value vector V through convolution. The similarity between the current pixel and all pixels in the feature map is then calculated to capture global dependencies. The similarity scores are used as weights to aggregate features from all spatial positions. In this way, spatially enhanced features with global semantics for the current pixel can be obtained, thereby overcoming the limitation of the convolutional receptive field. The detailed computation is as follows. First, the input feature map X is passed through 1×1 convolutions to generate Q, K, and V. Then, max pooling is applied, and the similarity between any two pixels is calculated. After the softmax activation function, the similarity matrix A between any two pixels is obtained. It is then multiplied by the value vector V to aggregate global information. Next, a 1×1 convolution is used to fuse the features and obtain $Y_{s}$. Finally, a residual connection is adopted to obtain the enhanced feature representation.

(15) $$Q=W_{q}\;X,\;K=W_{k}\;X,\;V=W_{v}\;X$$

(16) $$A=\mathrm{Softmax}(\mathrm{MaxPooling}(Q)K^{T})$$

(17) $$Y_{s}\;=W_{z}(\;AV)$$

(18) $$X_{s}=X+Y_{s}$$

$W_{q}$, $W_{k}$, and $W_{v}$ denote 1×1 convolutions, and MaxPooling(·) denotes max pooling. $W_{z}$ represents a 1×1 convolution, and $X_{s}$ denotes the output of the global spatial attention branch.

#### 2.3.2. Temporal Attention

For the temporal attention branch, the input feature map X is first processed by adaptive average pooling along the spatial dimensions to obtain the global response intensity of each channel. In this way, the model obtains a global vector $Z_{t}$ that describes the importance of each channel, namely each time frame. Then, two fully connected layers, together with ReLU and Sigmoid activation functions, are used to generate a set of normalized weight coefficients $S_{t}$. These coefficients are used to adaptively recalibrate the features of different time frames.

(19) $$Z_{t}=\mathrm{AdaptiveAvg}\mathrm{Pooling}(X)\in {\mathbb{R}}^{B\times C\times 1\times 1}$$

(20) $$S_{t}\;=\mathrm{Sigmoid}(W_{2}\mathrm{ReLU}(W_{1}Z_{t}))$$

AdaptiveAvgPooling(·) denotes adaptive average pooling, $W_{1}$ and $W_{2}$ denote the weights of the fully connected layers.

#### 2.3.3. Spatiotemporal Fusion Strategy

After obtaining the spatially enhanced features and temporally enhanced features, STFA integrates them with the original features through a gated fusion strategy. This produces the final spatiotemporally enhanced output features. The fusion process preserves the original feature information while modeling the spatiotemporal evolution patterns of radar echo sequences.

(21) $$Y=X⊗(S_{t}⊗X_{s})$$

⊗ denotes element-wise multiplication.

By introducing STFA into the skip connections, the model can fully exploit spatial detail information from different hierarchical levels during feature transmission. It can also dynamically perceive the evolutionary correlations of radar echoes along both temporal and spatial dimensions. This provides more discriminative spatiotemporal feature representations for high-quality reconstruction in the decoding stage.

## 3. Experimental Results and Analysis

### 3.1. Dataset

The proposed method was evaluated on two representative public radar datasets, namely SEVIR and CIKM.

- The SEVIR dataset [27] is a spatiotemporally aligned multimodal dataset. It contains more than 10,000 weather events. Each event consists of an image sequence covering a spatial region of 384 × 384 km over 4 h, with a total of 49 frames. In this study, precipitation storm events were used. For each weather event, a sliding window containing 25 consecutive frames was used to construct the samples. The starting indices of two adjacent sliding windows were separated by 12 frames; that is, the sampling stride of the sliding window was 12 frames. The 25 frames within each sample remained temporally consecutive and retained the original temporal resolution of 5 min. According to chronological order, the data were divided into three periods: 1 January 2017 to 31 December 2018, 1 January 2019 to 1 June 2019, and 2 June 2019 to 31 December 2019. Each sample contains 25 images with a temporal resolution of 5 min. Among them, 7490 sequences were used for training, 1221 sequences for validation, and 2262 sequences for testing. All sequences were uniformly downsampled to a spatial resolution of 128 × 128 pixels. Specifically, the first 5 frames were used as the model input, and the subsequent 20 frames were used as the target sequence.
- The CIKM AnalytiCup 2017 radar dataset records precipitation events in Guangdong, China, covering an area of 101 km × 101 km. It contains 14,000 consecutive radar echo sequence samples. Each sample consists of 15 consecutive radar echo maps with a temporal resolution of 6 min. Among them, 8000 sequences were used for training, 2000 sequences for validation, and 4000 sequences for testing. Each radar echo map was padded to 128 × 128 pixels. Specifically, the first 5 frames were used as the model input, and the subsequent 10 frames were used as the target sequence.

### 3.2. Evaluation Metrics

First, for the SEVIR dataset, the pixel values of all frames were remapped to the range of 0–255. To comprehensively evaluate the prediction performance under different rainfall intensities, the precipitation thresholds for this dataset were selected according to the six-level Video Integrator and Processor (VIP) intensity scale [28]. This scale is widely used in operational radar applications and corresponds to different precipitation intensity levels. Specifically, the pixel thresholds [16, 74, 133, 160, 181, 219] were selected to cover different intensity ranges from weak precipitation to strong convective precipitation. For the CIKM dataset, the pixel values in each frame were converted into reflectivity values within [0, 80] dBZ. To comprehensively evaluate the model under different rainfall intensities, four precipitation thresholds [20, 30, 35, 40] were used. Based on the thresholds selected for the two datasets, the predicted radar echo maps were compared with the observed radar echo maps for statistical evaluation. Specifically, if a pixel value was greater than a given precipitation threshold, it was set to 1; otherwise, it was set to 0. Then, the numbers of true positives (TP, prediction = 1 and ground truth = 1), false positives (FP, prediction = 1 and ground truth = 0), true negatives (TN, prediction = 0 and ground truth = 0), and false negatives (FN, prediction = 0 and ground truth = 1) were counted. Based on these statistics, the Critical Success Index (CSI), Heidke Skill Score (HSS), and Probability of Detection (POD) were calculated as evaluation metrics.

(22) $$CSI=\frac{TP}{TP+FN+FP}$$

(23) $$HSS=\frac{2(TP\times TN-FN\times FP)}{\left(TP+FN)(FN+TN)+(TP+FP)(FP+TN\right)}$$

(24) $$POD=\frac{TP}{TP+FN}$$

### 3.3. Experimental Settings

All experiments were conducted using the PyTorch 2.3.1 framework on an NVIDIA GeForce RTX 4090 GPU (NVIDIA Corporation, Santa Clara, CA, USA). The batch size was set to 4, and the Adam optimizer with an initial learning rate of 0.0001 was used for optimization. The number of training epochs was set to 100. In addition, mean squared error (MSE) was used as the loss function. To prevent model overfitting, early stopping was adopted for all networks during training.

### 3.4. Comparative Experiments

To validate the effectiveness of the proposed ST-TriMambaUNet, systematic comparisons were conducted with nine representative methods. These methods include ConvLSTM [8], which learns temporal dependencies with a recurrent structure; a series of UNet-based methods, including SmaAt-UNet [16], SAR-UNet [17], and SSA-UNet [18]; and a series of Transformer-based methods, including AA-TransUNet [20], Rainformer [21], RainHCNet [24], LPT-QPN [29], and Earthformer [30]. All comparison models were implemented using their publicly available source codes. The experimental results on the SEVIR dataset are shown in Table 1, Table 2 and Table 3, while those on the CIKM AnalytiCup 2017 dataset are shown in Table 4, Table 5 and Table 6. The column headers 16, 74, 133, 160, 181, and 219 denote the pixel-value thresholds of the SEVIR data after remapping to the range of 0–255. These thresholds were selected according to the six-level VIP intensity scale and represent precipitation intensities ranging from weak precipitation to strong convective precipitation. They are not reflectivity values in dBZ. The best results are highlighted in bold, and the second-best results are underlined. The symbol ↑ indicates that a higher value represents better performance.

As shown in Table 1, Table 2 and Table 3, the proposed ST-TriMambaUNet achieved the best or second-best performance under most thresholds for the three metrics. Its average performance was significantly better than that of existing methods. Specifically, the mean CSI, POD, and HSS of the proposed model reached 0.3535, 0.4326, and 0.4479, respectively. Compared with LPT-QPN, which achieved the second-best average performance, these values were improved by 2.08%, 1.67%, and 2.45%, respectively. This indicates that the proposed model has an advantage in overall prediction accuracy. In terms of performance under different precipitation thresholds, ST-TriMambaUNet performed particularly well in heavy rainfall scenarios. Specifically, when the precipitation thresholds were 160, 181, and 219, the CSI values of the proposed model reached 0.2387, 0.1838, and 0.0780, respectively. Compared with the second-best results under the corresponding thresholds, they were improved by 4.92%, 10.19%, and 7.29%, respectively. For POD, the proposed model achieved 0.2869, 0.2176, and 0.0915 under the thresholds of 160, 181, and 219, respectively. These values were 6.02%, 12.22%, and 4.93% higher than the second-best results. For HSS, the proposed model reached 0.3563, 0.2843, and 0.1263 under the three high thresholds, respectively. These values exceeded the second-best results by 4.98%, 10.97%, and 6.40%, respectively. Overall, although the proposed model did not always achieve the best results under some low thresholds, it showed stronger competitiveness in the more challenging high-threshold heavy rainfall scenarios. It achieved better performance on the three key metrics, namely CSI, POD, and HSS. These results demonstrate that the proposed method can effectively improve the prediction ability for high-intensity precipitation events.

As shown in Table 4, Table 5 and Table 6, the proposed ST-TriMambaUNet achieved the best or second-best CSI, HSS, and POD under most thresholds. Considering the average results of the three metrics under the four precipitation thresholds, the mean CSI, POD, and HSS of the proposed model reached 0.3091, 0.3848, and 0.4012, respectively. Among them, the mean CSI and mean HSS achieved the best results, exceeding the corresponding second-best results by 2.05% and 2.79%, respectively. The mean POD achieved the second-best result and was only lower than that of ConvLSTM. As shown in Table 5, under the low thresholds of 20 dBZ and 30 dBZ, the POD values of the proposed model were 0.7352 and 0.3646, respectively. These values were lower than those of ConvLSTM, which were 0.7676 and 0.4820. This indicates that the proposed model has relatively weaker recall ability for weak precipitation regions under low-threshold conditions. This is because POD mainly measures the model’s recall ability for precipitation events. In low-threshold scenarios, a higher POD often means that the model tends to identify more weak precipitation regions. However, this may also introduce a higher risk of false alarms. The proposed model focuses more on improving the prediction accuracy for heavy rainfall. Therefore, it produces more conservative predictions for weak precipitation regions. Although this strategy helps reduce false alarms to some extent, it also leads to a slightly lower POD under low thresholds. This can also be supported by the visualization results in Section 3.5. Compared with the proposed model, ConvLSTM is more likely to generate echoes over a larger area and severely underestimates heavy precipitation regions. In contrast, the proposed model tends to preserve the structure of the main rainband and the strong-echo core regions. It also predicts the marginal low-intensity regions more conservatively. This suggests that the proposed model is more inclined to highlight high-intensity precipitation regions rather than expand the coverage of weak precipitation. This phenomenon is consistent with the quantitative results in Table 5. Under low thresholds, ConvLSTM can more easily obtain a higher POD by expanding the coverage of weak precipitation regions. By contrast, the proposed model emphasizes the discrimination and preservation of heavy rainfall core regions. Therefore, it produces relatively conservative predictions under low-threshold scenarios, resulting in a POD slightly lower than that of ConvLSTM.

In terms of performance under different precipitation thresholds, the proposed model showed more obvious advantages in heavy rainfall scenarios. Specifically, when the threshold was 35 dBZ, the proposed model achieved the best results in CSI, POD, and HSS, reaching 0.2154, 0.2691, and 0.3213, respectively. These values exceeded the corresponding second-best results by 7.00%, 1.97%, and 6.18%, respectively. When the threshold was 40 dBZ, the proposed model also achieved the best results in all three metrics, reaching 0.1374, 0.1701, and 0.2140, respectively. These values were 5.29%, 8.07%, and 4.65% higher than the corresponding second-best results. This indicates that under more challenging high-threshold precipitation conditions, ST-TriMambaUNet can more accurately identify strong-echo regions and more effectively preserve the structural features of heavy rainfall.

**Figure 5:**
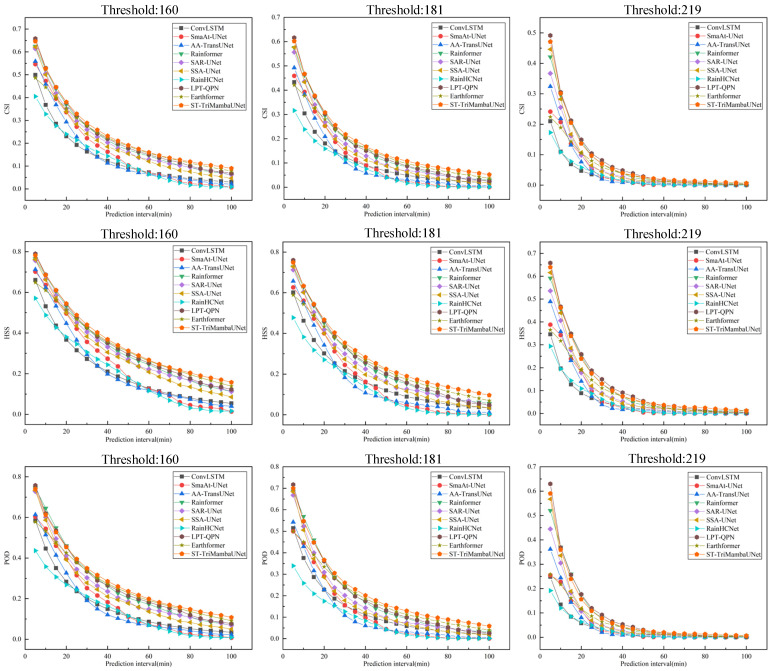
CSI, HSS, and POD curves of different models on the SEVIR dataset at thresholds of 160, 181, and 219.

**Figure 6:**
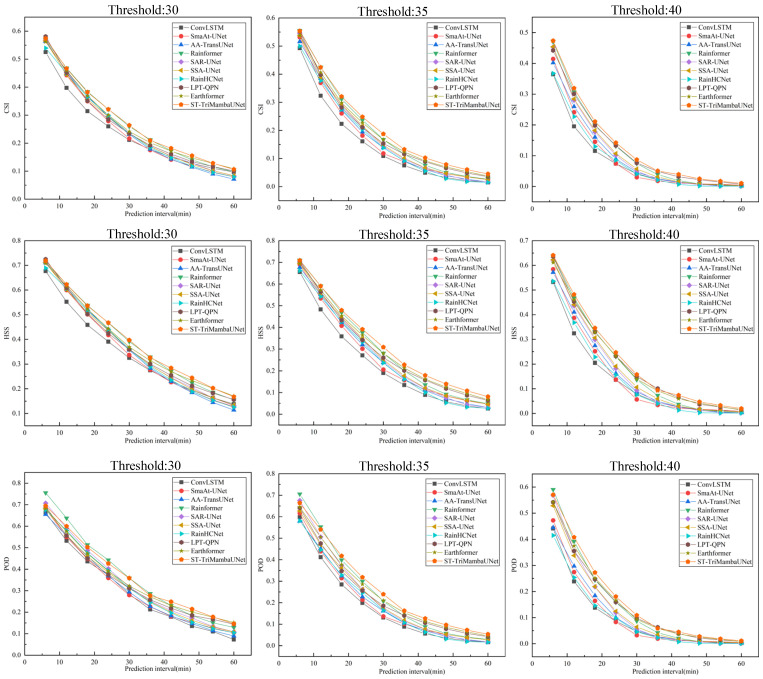
CSI, HSS, and POD curves of different models on the CIKM dataset at thresholds of 30, 35, and 40.

To facilitate the observation of how the prediction performance of different models changes as the lead time increases, and to further evaluate their ability to capture temporal dependencies over long time steps. CSI, POD, and HSS curves were plotted. Specifically, for the SEVIR dataset, the curves were drawn under the three heavy rainfall thresholds of 160, 181, and 219. For the CIKM AnalytiCup 2017 dataset, the curves were drawn under the three heavy rainfall thresholds of 30, 35, and 40. The corresponding results are shown in Figure 5 and Figure 6.

As shown in Figure 5, under the heavy rainfall thresholds of the SEVIR dataset, the CSI, HSS, and POD of all models gradually decreased as the lead time increased. This indicates that the difficulty of forecasting heavy rainfall increases significantly with longer prediction horizons. In particular, when the threshold was 219, the three metrics of all models rapidly approached zero in the later prediction stage. This suggests that long-term prediction remains highly challenging under extremely heavy rainfall conditions.

A further horizontal comparison among different models shows that the proposed ST-TriMambaUNet maintained better performance at most prediction time steps. Its overall decreasing trend was also relatively smoother. Under the thresholds of 160 and 181, the curves of the proposed model were generally above those of most comparison models in terms of CSI, HSS, and POD. In particular, it still maintained relatively high metric values in the middle and late prediction stages. This indicates that the proposed model has better temporal dependency modeling ability and more stable prediction performance under heavy rainfall conditions. This advantage also remained under the higher rainfall threshold of 219. Although all models showed obvious degradation in the later prediction stage, the proposed model still maintained a leading position at most time steps. This demonstrates its stronger ability to identify and preserve high-intensity echo regions in extreme heavy rainfall scenarios.

It can also be observed that, compared with some comparison models, the proposed model achieved higher metric values in the early prediction stage. Moreover, its performance degraded more slowly as the lead time increased. This indicates that ST-TriMambaUNet can effectively capture the spatial structural features of short-term heavy rainfall regions. It can also better preserve the information of the main rainband and strong-echo core regions during long-term prediction. As a result, it alleviates the performance degradation caused by error propagation over time.

Overall, the curves in Figure 5 further demonstrate that the proposed ST-TriMambaUNet has more obvious advantages in heavy rainfall scenarios on the SEVIR dataset. In particular, under high-threshold conditions and long-term prediction tasks, it can more effectively maintain the prediction ability for heavy rainfall regions. This shows that the proposed method is highly competitive in heavy rainfall prediction and temporal dependency modeling.

As shown in Figure 6, as the prediction interval increased, the CSI, HSS, and POD of all models showed a continuous downward trend under high rainfall thresholds on the CIKM dataset. This indicates that accurately characterizing heavy rainfall regions becomes more difficult in long-term prediction tasks. Therefore, precipitation nowcasting under high-threshold scenarios is more challenging.

A further comparison of the curves of different models shows that the proposed ST-TriMambaUNet maintained better performance at most prediction time steps. Its overall decreasing trend was also relatively smoother. When the thresholds were 35 and 40 dBZ, the curves of the proposed model for CSI, HSS, and POD were above those of all comparison models at all time steps. In particular, it still maintained relatively high metric values in the middle and late prediction stages. This indicates that the proposed model has better temporal dependency modeling ability and more stable prediction performance in heavy rainfall scenarios. This advantage remained evident under the higher rainfall threshold of 40 dBZ.

In addition, the curve trends show that the proposed model achieved high metric values in the early prediction stage. Its performance also degraded more slowly as the lead time increased. Compared with some comparison models that showed more obvious performance degradation in the later stages, ST-TriMambaUNet maintained more stable CSI, HSS, and POD over a longer prediction range. This indicates that the proposed method can effectively capture the spatial structural features of short-term heavy rainfall regions. It can also better preserve temporal evolution information during long-term prediction.

Figure 5 and Figure 6 evaluate the temporal dynamics learned by different models based on quantitative results at different forecast lead times. Since CSI, HSS, and POD are calculated independently for each predicted frame, their variations with forecast lead time reflect the ability of a model to preserve temporal evolution information during continuous prediction. For the SEVIR dataset, adjacent predicted frames are separated by 5 min, resulting in a forecast range of 5–100 min. The proposed model maintained a clear relative advantage under the thresholds of 160 and 181, particularly from 40 to 100 min, while its advantage under the threshold of 219 was mainly concentrated within 5–40 min. For the CIKM dataset, adjacent predicted frames are separated by 6 min, resulting in a forecast range of 6–60 min. The proposed model remained competitive throughout the 6–60 min forecast range under the thresholds of 35 and 40 dBZ, with the most evident advantage occurring between 18 and 48 min.

### 3.5. Visualization Analysis

**Figure 7:**
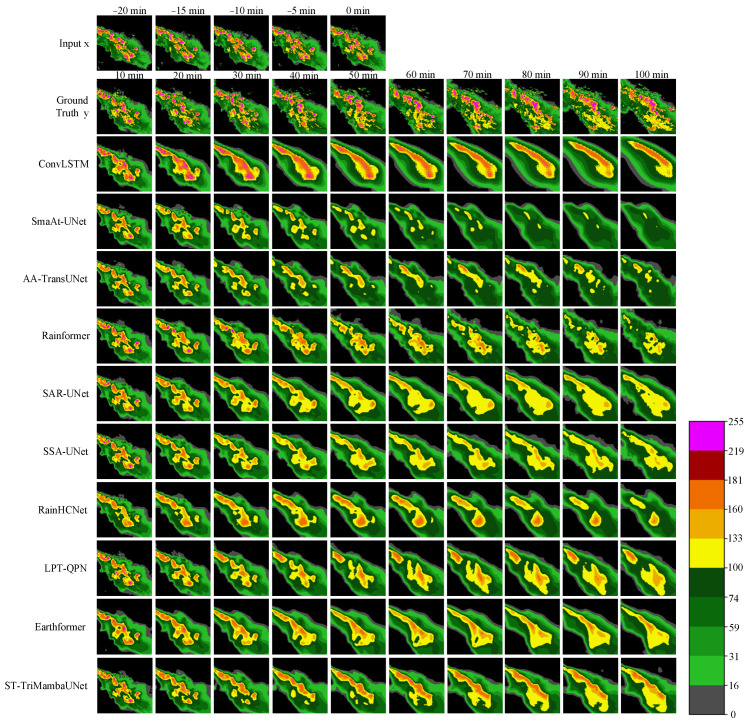
Visualization results of different models on the SEVIR dataset.

**Figure 8:**
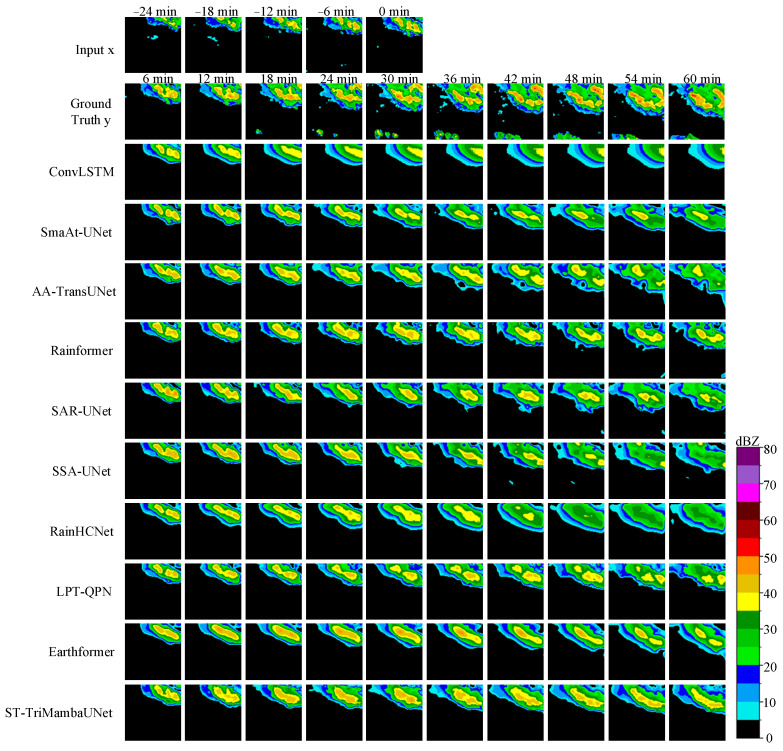
Visualization results of different models on the CIKM dataset.

To more intuitively compare the prediction performance of different models, one precipitation event sample was selected from the SEVIR and CIKM datasets, respectively. The prediction results of all models were then visually compared, as shown in Figure 7 and Figure 8.

As further observed from Figure 7, all models could capture the overall motion trend, but most of them showed obvious degradation in long-term prediction. As the lead time increased, the predicted heavy rainfall regions of ConvLSTM became stretched in shape. SmaAt-UNet and AA-TransUNet could well reflect the main precipitation distribution in the short-term prediction stage. However, they showed clear intensity underestimation and morphological shrinkage in the middle and late stages. Rainformer could locate the strong-echo core relatively well, but its predictions often suffered from precipitation-range displacement and discontinuous echo morphology during temporal evolution. Other models, such as SAR-UNet, SSA-UNet, and RainHCNet, also showed echo smoothing and local discontinuities to varying degrees. LPT-QPN preserved the overall contour of the rainband relatively well in the early and middle stages. It also showed a certain ability to maintain the strong-echo core regions. However, as the lead time increased, its predictions still gradually suffered from intensity attenuation. Local discontinuities also appeared, especially at long time steps. In contrast, Earthformer was relatively stable in preserving the overall spatial distribution and the main rainband morphology. Nevertheless, it also showed some weakening of strong-echo regions in the middle and late prediction stages. Its ability to preserve local high-intensity precipitation cores was weaker than that of the proposed method.

By comparison, the proposed ST-TriMambaUNet showed clear advantages. Even at a long lead time of 100 min, it could still maintain the complete morphology of the rainband. The spatial location and intensity distribution of the strong-echo regions were consistent with the observed results. No obvious morphological collapse or intensity underestimation was observed. Overall, ST-TriMambaUNet effectively alleviated the common problems of morphological drift and intensity attenuation observed in other models. The generated predictions were closer to the real precipitation evolution process.

As shown in Figure 8, as the lead time increased, all models suffered from information loss and intensity attenuation in heavy rainfall regions to varying degrees. Although ConvLSTM could capture temporal dependencies relatively well, it showed obvious prediction bias and large-scale loss of rainfall information. As a result, it was difficult for ConvLSTM to predict the overall morphological structure of the rainband. Compared with other models, Rainformer predicted heavy rainfall regions more effectively. However, the morphological structure of the rainband was clearly lost. LPT-QPN preserved the overall contour of the rainband well in the early prediction stage and located the main precipitation regions accurately. Nevertheless, as the lead time increased, its predictions gradually showed echo discontinuities and underestimation of heavy rainfall regions. In contrast, Earthformer was relatively stable in preserving the overall spatial distribution and main rainband morphology. However, it also suffered from attenuation in heavy rainfall regions in the middle and late prediction stages. Its characterization of local high-intensity echo cores was still less sufficient than that of the proposed model. By comparison, the proposed ST-TriMambaUNet more accurately maintained the overall spatial distribution and evolution trend of the rainband and significantly reduced intensity attenuation. This is highly consistent with the conclusions drawn from the quantitative metrics, further validating the effectiveness of the proposed method. It should also be noted that none of the models successfully predicted the second rainband appearing in the later observations. This rainband was not clearly present in the five historical input frames. Since each CIKM sample covers a limited spatial region of approximately 101 km × 101 km, the rainband may have moved into the study area from outside the observed domain, or it may have developed locally during the forecasting period. Unlike the information loss of existing precipitation features during long-term prediction, the missed second rainband may be mainly attributed to the absence of its contextual echo information in the model input. This result further demonstrates the limitation of deterministic radar echo extrapolation models in predicting precipitation systems that are not represented in the historical input sequence.

In summary, both quantitative metrics and qualitative analysis show that ST-TriMambaUNet has clear advantages in terms of overall performance and in heavy rainfall thresholds. These results verify its superiority in precipitation nowcasting tasks.

### 3.6. Ablation Study

To evaluate the contributions of the proposed STFA and MSIM modules to the overall performance, ablation studies were conducted on the CIKM dataset. The training configuration was kept consistent with that used in the comparative experiments, and only the key components of the model were replaced or removed. Five model variants were constructed: (1) removing STFA, (2) replacing MSIM with a standard 3×3 convolutional block, (3) removing the original Mamba branch from MSIM, (4) removing the Mamba branch with channel shuffle, and (5) removing the Mamba branch with multi-scale directional convolution. The experimental results are shown in Table 7. All metrics were evaluated under two heavy rainfall thresholds, namely 35 dBZ and 40 dBZ, using the meteorological metrics CSI, POD, and HSS.

As shown in Table 7, the complete model achieved the best results under all thresholds and significantly outperformed the other variants. When STFA was removed, under the threshold of 35 dBZ, CSI decreased to 0.2049, POD decreased to 0.2478, and HSS decreased to 0.3073. Furthermore, after different branches in the MSIM module were progressively removed, a more obvious performance degradation was observed. Under the threshold of 35 dBZ, after removing the Mamba branch, CSI decreased from 0.2154 to 0.2108, and HSS decreased from 0.3213 to 0.3151. When the Mamba branch with channel shuffle was further removed, CSI decreased to 0.2099, and HSS decreased to 0.3143. When the Mamba branch with multi-scale directional convolutions was also removed, the most significant performance degradation occurred. CSI decreased to 0.2086, and HSS decreased to 0.312. These results indicate that the multi-scale directional convolution branch and the channel-shuffle branch contributed most to the model performance. The former improved the extraction of directional and scale-varying features, while the latter enhanced cross-channel information interaction. Both branches played key roles in identifying heavy rainfall regions. Overall, removing any branch of the MSIM module led to obvious performance degradation, while the complete model achieved the best performance. This validates the effectiveness of MSIM and STFA in improving the prediction accuracy for heavy rainfall. These results further demonstrate that the three branches learn complementary feature representations. Unlike an early-fusion design that combines different types of features before sufficient branch-specific learning, the proposed MSIM module first employs three specialized branches to independently model global spatiotemporal dependencies, cross-channel feature interactions, and multi-scale directional structures, and subsequently performs adaptive fusion of their outputs. Premature fusion may weaken the functional specialization of the three branches and cause different types of information to interfere with one another during subsequent shared processing. In contrast, the proposed adaptive gated fusion mechanism dynamically adjusts the contributions of each branch according to the local spatiotemporal features at different spatial locations, thereby preserving and selectively integrating their complementary information. In addition, because temporal information has already been embedded into the channel representation before entering the MSIM module, the fusion coefficients cannot be directly interpreted as branch weights at individual forecast lead times.

**Figure 9:**
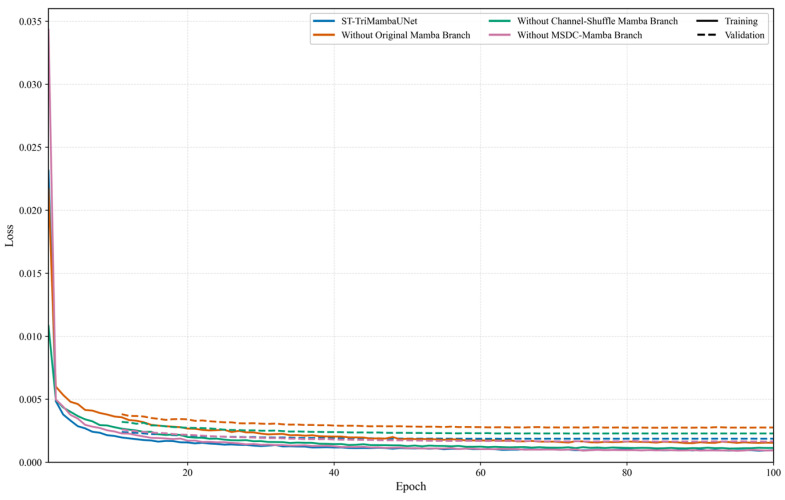
Training and validation loss curves of ST-TriMambaUNet and its branch-ablation variants.

To further investigate the effects of the three MSIM branches on model convergence and training stability, the training and validation loss curves of the complete ST-TriMambaUNet and its three branch-ablation variants are compared in Figure 9.

Figure 9 presents the training and validation loss curves of ST-TriMambaUNet and its three branch-removal variants. The training losses of all models decreased rapidly during the early training stage and gradually stabilized as the number of epochs increased. Meanwhile, the validation losses followed similar decreasing trends without a persistent increase, indicating stable convergence and no obvious overfitting. Removing the original Mamba branch or the channel-shuffle Mamba branch resulted in higher training and validation losses, demonstrating their contributions to global spatiotemporal modeling and cross-channel feature interaction. The model without the MSDC-Mamba branch achieved a slightly lower pixel-wise loss.

## 4. Conclusions

This paper proposes ST-TriMambaUNet, a weather-radar-echo-extrapolation-based spatiotemporal sequence prediction network for precipitation nowcasting. Based on the U-Net framework, a Spatiotemporal Fusion Attention (STFA) and a Multi-Scale Interaction Mamba (MSIM) module were designed. STFA consists of global spatial attention and temporal attention. It can effectively capture long-range spatial correlations and model temporal dependencies among radar echoes in a parallel manner. The MSIM module consists of an original Mamba branch, a Mamba branch with channel shuffle, and a multi-scale, directionally-convolutional Mamba branch. It further enhances spatiotemporal feature interaction among different time frames. It also learns the multi-scale directional features of heavy rainfall regions, thereby improving the prediction accuracy for heavy rainfall. Extensive experiments on the CIKM and SEVIR datasets showed that the proposed method significantly outperformed several representative methods.

Although ST-TriMambaUNet can significantly improve the prediction accuracy for heavy rainfall, it still has some limitations. The proposed model is a deterministic model. When mean squared error (MSE) is used as the loss function for optimization, the model tends to average multiple possible future states. This may produce over-smoothed and blurry prediction results. Therefore, the local fidelity of radar echoes still needs to be further improved. Future research can be carried out from the following aspects. First, this study mainly modeled radar echo data. In the future, multimodal data such as numerical weather prediction products and satellite observations could be incorporated. Second, generative modeling could be introduced. Precipitation nowcasting can be formulated as a conditional probability distribution modeling problem. This may alleviate the over-smoothing problem and better meet the needs of precipitation nowcasting. Furthermore, neighborhood-based verification metrics, such as the Fractions Skill Score, will be evaluated under multiple precipitation thresholds and spatial neighborhood sizes to more comprehensively assess the displacement errors and the structural consistency of the predicted rainbands.

## Figures and Tables

**Table 1:** CSI results of different models on the SEVIR dataset.

Model	*CSI* ↑						
	16	74	133	160	181	219	AVG
ConvLSTM	0.5827	0.4912	0.2066	0.1359	0.1046	0.0298	0.2585
SmaAt-UNet	0.6861	0.5779	0.2508	0.1607	0.1086	0.0403	0.3041
AA-TransUNet	0.6761	0.5755	0.2482	0.1476	0.1013	0.0431	0.2986
Rainformer	0.6840	0.5941	0.3231	0.2193	0.1614	0.0727	0.3424
SAR-UNet	0.6821	**0.6013**	0.3223	0.2107	0.1476	0.0574	0.3369
SSA-UNet	0.6790	0.5912	0.3096	0.1979	0.1388	0.0630	0.3299
RainHCNet	0.6748	0.5631	0.2206	0.1250	0.0749	0.0274	0.2810
LPT-QPN	0.6766	0.5987	**0.3376**	0.2275	0.1668	0.0703	0.3463
Earthformer	0.6759	0.5894	0.3204	0.2170	0.1572	0.0508	0.3351
ST-TriMambaUNet	**0.6878**	0.5964	0.3362	**0.2387**	**0.1838**	**0.0780**	**0.3535**

Note: ↑ indicates that a higher value represents better performance. Bold values indicate the best results, and underlined values indicate the second-best results.

**Table 2:** POD results of different models on the SEVIR dataset.

Model	*POD* ↑						
	16	74	133	160	181	219	AVG
ConvLSTM	0.8795	0.5935	0.2530	0.1633	0.1280	0.0358	0.3422
SmaAt-UNet	0.8512	0.6763	0.2914	0.1813	0.1205	0.0435	0.3607
AA-TransUNet	0.8585	0.6775	0.2819	0.1616	0.1103	0.0472	0.3561
Rainformer	0.8548	0.7263	0.4120	0.2660	0.1920	0.0872	0.4231
SAR-UNet	0.8791	0.7293	0.3975	0.2456	0.1694	0.0659	0.4144
SSA-UNet	0.8673	0.7085	0.3766	0.2277	0.1572	0.0740	0.4019
RainHCNet	0.8536	0.6517	0.2539	0.1386	0.0816	0.0300	0.3349
LPT-QPN	**0.8807**	**0.7319**	0.4197	0.2706	0.1939	0.0863	0.4255
Earthformer	0.8646	0.7209	0.4085	0.2619	0.1853	0.0565	0.4163
ST-TriMambaUNet	0.8562	0.7195	**0.4238**	**0.2869**	**0.2176**	**0.0915**	**0.4326**

Note: ↑ indicates that a higher value represents better performance. Bold values indicate the best results, and underlined values indicate the second-best results.

**Table 3:** HSS results of different models on the SEVIR dataset.

Model	*HSS* ↑						
	16	74	133	160	181	219	AVG
ConvLSTM	0.6222	0.6065	0.3068	0.2145	0.1708	0.0533	0.3290
SmaAt-UNet	0.7452	0.6903	0.3592	0.2434	0.1699	0.0693	0.3796
AA-TransUNet	0.7336	0.6875	0.3615	0.2267	0.1596	0.0718	0.3735
Rainformer	0.7420	0.7030	0.4543	0.3289	0.2496	0.1184	0.4327
SAR-UNet	0.7383	**0.7095**	0.4549	0.3190	0.2310	0.0956	0.4247
SSA-UNet	0.7358	0.7009	0.4390	0.2998	0.2163	0.1015	0.4155
RainHCNet	0.7345	0.6778	0.3318	0.2005	0.1258	0.0497	0.3534
LPT-QPN	0.7316	0.7061	**0.4712**	0.3394	0.2562	0.1187	0.4372
Earthformer	0.7347	0.6998	0.4563	0.3339	0.2529	0.0899	0.4279
ST-TriMambaUNet	**0.7462**	0.7045	0.4699	**0.3563**	**0.2843**	**0.1263**	**0.4479**

Note: ↑ indicates that a higher value represents better performance. Bold values indicate the best results, and underlined values indicate the second-best results.

**Table 4:** CSI results of different models on the CIKM dataset.

Model	*CSI* ↑				
	20 dBZ	30 dBZ	35 dBZ	40 dBZ	AVG
ConvLSTM	0.5802	0.2419	0.1842	0.1071	0.2784
SmaAt-UNet	0.5984	0.2467	0.1717	0.0954	0.2780
AA-TransUNet	0.5922	0.2510	0.1741	0.1007	0.2795
Rainformer	**0.6121**	0.2740	0.2001	0.1254	0.3029
SAR-UNet	0.6000	0.2574	0.1830	0.1108	0.2878
SSA-UNet	0.5922	0.2546	0.1856	0.1147	0.2868
RainHCNet	0.5962	0.2511	0.1701	0.0880	0.2763
LPT-QPN	0.6073	0.2606	0.1950	0.1305	0.2984
Earthformer	0.6024	0.2688	0.2013	0.1274	0.3000
ST-TriMambaUNet	0.6049	**0.2789**	**0.2154**	**0.1374**	**0.3091**

Note: ↑ indicates that a higher value represents better performance. Bold values indicate the best results, and underlined values indicate the second-best results.

**Table 5:** POD results of different models on the CIKM dataset.

Model	*POD*↑				
	20 dBZ	30 dBZ	35 dBZ	40 dBZ	AVG
ConvLSTM	**0.7676**	**0.4820**	0.2639	0.1346	**0.4120**
SmaAt-UNet	0.7251	0.3106	0.2011	0.1080	0.3362
AA-TransUNet	0.7192	0.3093	0.2010	0.1130	0.3356
Rainformer	0.7469	0.3689	0.2517	0.1550	0.3806
SAR-UNet	0.7291	0.3379	0.2266	0.1365	0.3575
SSA-UNet	0.7233	0.3246	0.2209	0.1342	0.3507
RainHCNet	0.7254	0.3259	0.2003	0.0991	0.3377
LPT-QPN	0.7389	0.3356	0.2330	0.1547	0.3655
Earthformer	0.7296	0.3475	0.2486	0.1574	0.3708
ST-TriMambaUNet	0.7352	0.3646	**0.2691**	**0.1701**	0.3848

Note: ↑ indicates that a higher value represents better performance. Bold values indicate the best results, and underlined values indicate the second-best results.

**Table 6:** HSS results of different models on the CIKM dataset.

Model	*HSS* ↑				
	20 dBZ	30 dBZ	35 dBZ	40 dBZ	AVG
ConvLSTM	0.6410	0.3373	0.2795	0.1751	0.3582
SmaAt-UNet	0.6676	0.3544	0.2601	0.1511	0.3583
AA-TransUNet	0.6603	0.3595	0.2632	0.1602	0.3608
Rainformer	**0.6804**	0.3897	0.2970	0.1940	0.3903
SAR-UNet	0.6687	0.3677	0.2733	0.1722	0.3705
SSA-UNet	0.6601	0.3653	0.2789	0.1789	0.3708
RainHCNet	0.6653	0.3604	0.2573	0.1421	0.3563
LPT-QPN	0.6752	0.3724	0.2935	0.2045	0.3864
Earthformer	0.6708	0.3848	0.3026	0.2005	0.3897
ST-TriMambaUNet	0.6731	**0.3966**	**0.3213**	**0.2140**	**0.4012**

Note: ↑ indicates that a higher value represents better performance. Bold values indicate the best results, and underlined values indicate the second-best results.

**Table 7:** Ablation results on the CIKM dataset.

MSIM	STFA	*CSI* ↑		*POD* ↑		*HSS* ↑	
		35 dBZ	40 dBZ	35 dBZ	40 dBZ	35 dBZ	40 dBZ
Full MSIM	Disabled	0.2049	0.1276	0.2478	0.1528	0.3073	0.1993
MSIM replaced by a 3 × 3 convolutional block	Enabled	0.2043	0.1227	0.2453	0.1447	0.3062	0.1912
Without the original Mamba branch	Enabled	0.2108	0.1336	0.2594	0.1630	0.3151	0.2081
Without channel-shuffle Mamba branch	Enabled	0.2099	0.1319	0.2572	0.1599	0.3143	0.2063
Without MSDC-Mamba branch	Enabled	0.2086	0.1317	0.2549	0.1597	0.3124	0.2054
Full MSIM	Enabled	**0.2154**	**0.1374**	**0.2691**	**0.1701**	**0.3213**	**0.2140**

Note: ↑ indicates that a higher value represents better performance. Bold values indicate the best results.

## Data Availability

The dataset is available at https://registry.opendata.aws/sevir/(SEVIR) (accessed on 1 September 2025) and https://tianchi.aliyun.com/dataset/1085(CIKM) (accessed on 1 September 2025).

## Abbreviations

The following abbreviations are used in this manuscript:

ST-TriMambaUNet	Spatiotemporal Fusion Attention-enhanced Tri-branch MambaUNet
STFA	Spatiotemporal Fusion Attention
MSIM	Multi-Scale Interaction Mamba
MSDC	Multi-Scale Directional Convolution
